# CD4^+^FoxP3^+^ Regulatory T Cells from Gαi2^−/−^ Mice Are Functionally Active *In Vitro*, but Do Not Prevent Colitis

**DOI:** 10.1371/journal.pone.0025073

**Published:** 2011-09-22

**Authors:** Yu-Yuan C. Götlind, Sukanya Raghavan, Paul W. Bland, Elisabeth Hultgren Hörnquist

**Affiliations:** 1 Department of Microbiology & Immunology, Institute of Biomedicine, Mucosal Immunology and Vaccine Center, University of Gothenburg, Gothenburg, Sweden; 2 University of Bristol, Clinical Veterinary Science, Bristol, United Kingdom; 3 School of Health and Medical Sciences, Örebro University, Örebro, Sweden; Centre de Recherche Public de la Santé (CRP-Santé), Luxembourg

## Abstract

**Background:**

Mice deficient in the inhibitory G protein subunit Gαi2 spontaneously develop a T helper 1 dominated colitis. We examined whether a defect in CD4^+^FoxP3^+^ regulatory T cells (Treg) underpins the pathogenesis of colitis in the Gαi2^−/−^ (Gαi2-deficient) colitis model.

**Methodology/Principal Findings:**

Using flow cytometry, we found that thymus and colonic lamina propria, but not spleen and mesenteric lymph nodes, of colitic Gαi2^−/−^ mice contained increased frequencies of Treg, whereas FoxP3 expression intensity was similar in Gαi2^−/−^ compared to Gαi2^+/−^ or Gαi2^+/+^ wild type (WT) mice. The frequency of CD4^+^FoxP3^+^ T cells expressing CD103 was significantly increased in Gαi2^−/−^ compared to WT mice. Treg in colons from WT mice clustered in the T cell areas of colonic lymphoid patches (CLP), with relatively few Treg in the lamina propria, as demonstrated by immunohistochemistry. In Gαi2^−/−^ mice, CLP were not observed but lamina propria Treg were increased in number and frequency within the CD4^+^ infiltrate, compared to WT mice. Using an in vitro co-culture system and flow cytometric analysis of cell division we could demonstrate that the in vitro suppressive function of WT and Gαi2^−/−^ CD4^+^FoxP3^+^ regulatory T cells (WT-Treg and KO-Treg) was indistinguishable, but that T effector cells (CD4^+^25^−^ T cells) from Gαi2^−/−^ mice were less readily suppressed than WT effectors (WT-Teff) by Treg from either source. However, neither WT nor Gαi2^−/−^ Treg was able to suppress colitis induced by adoptive transfer of Gαi2^−/−^ effector T cells (KO-Teff) to RAG2^−/−^ recipients. The enhanced inflammatory activity of Gαi2^−/−^ effectors was accompanied by increased expression of an effector/memory T cell phenotype and increased cytokine secretion, especially IL-4, IL-6 and IFN-γ.

**Conclusions:**

There is an increased frequency of Gαi2^−/−^ Treg in the colon, and they demonstrate no endogenous functional defect. However, Gαi2^−/−^ T effector cells are dramatically less susceptible to suppression in vitro, and in vivo, despite increased effective numbers of Treg, they cannot prevent disease.

## Introduction

The mucosal immune dysregulation which results in chronic inflammatory bowel disease (IBD) has been evaluated using several chemically-induced, T cell transfer and spontaneous mouse models of colitis. Untreated Gαi2^−/−^ mice on a 129SvEv background spontaneously develop colitis [1], [2], [3]. The inflammation is restricted to the large intestine, resembling the pathology seen in human ulcerative colitis (UC) with crypt distortion, loss of mucin producing goblet cells, and abscess formation together with erosion and ulcerations of the mucosa [1], [2], [3].

The development of colitis in the Gαi2^−/−^ mouse model is dependent on the presence of an enteric microflora (unpublished observations) and is characterized by a Th1 CD4^+^ T cell response, with increased production of IFN-γ, together with increased levels of IL-1, IL-6 and TNF-α in inflamed tissue [1], [2], [4]. The lack of Gαi2 results in functional disorders in epithelial cells [5], professional antigen-presenting cells [6], B cells [2], [4], [6] and CD4^+^ T cells [1], [3], [7], [8], [9] and points to multiple requirements for Gαi2 in mucosal immune regulation, but the underlying molecular mechanisms involving Gαi2 have not been defined.

Mucosal tolerance to environmental and food antigens is essential to prevent exacerbated immune reactions that can cause allergic diseases and chronic inflammation, and it has long been recognized that the gut is an important site for the induction of tolerance [10]. Peripheral immune tolerance is mediated by several mechanisms, including anergy; deletion; ignorance; or suppression of effector T cell (Teff) function by different populations of regulatory T cells (Treg) [11]. There are two subsets of Treg: natural Treg (nTreg), which develop in the thymus; and inducible Treg (iTreg), which are derived from naïve CD4^+^ T cells in the periphery. Inducible Treg comprise type 1 regulatory T cells (Tr1), which are induced by IL-10 [12] and T helper 3 (Th3) cells, which are induced by TGFβ [13]. Treg utilize a variety of mechanisms to suppress the immune response. Generally, nTreg are believed to suppress by a cell–cell contact-dependent mechanism in vitro (possibly by granzyme or perforin-mediated cytotoxicity or by cAMP), while their regulatory effects in vivo are assumed to be mainly cytokine-mediated [14], [15].

The development, function, and homeostasis of Treg are controlled by the forkhead family transcription factor, Foxp3 [16], [17], [18]. Mutations in the gene encoding Foxp3/FOXP3 have been identified in both mice and humans [16], [19]. Patients with mutations in *FOXP3* develop a severe, fatal systemic autoimmune disorder, Immune Dysregulation Polyendocrinopathy Enteropathy X-linked (IPEX) syndrome [19]. Experiments using adoptive transfer of effector and regulatory T cell populations into immunodeficient mice indicate a crucial role for Treg in maintaining mucosal immune homeostasis [20], [21], suggesting that dysfunction of Treg may be a contributory factor in the pathogenesis of human IBD.

In this study, we address the hypothesis that colitis in Gαi2^−/−^ mice is a consequence of a qualitative or quantitative defect in CD4^+^FoxP3^+^ regulatory T cells (Treg). The data show that although Treg from Gαi2^−/−^ mice are functional in vitro, and are present at enhanced frequency within the CD4^+^ infiltrate in colitis, they are unable to prevent disease caused by endogenously activated Gαi2^−/−^ effector T cells, the latter being significantly more resistant to suppression by either wild type or Gαi2^−/−^ Treg.

## Materials and Methods

### Ethics statement

All animal procedures were carried out under local and national ethical guidelines (Swedish Board of Agriculture) and were approved by the regional ethical committee, Gothenburg Administrative Court of Appeal, with the ethical approval ID 79-2008.

### Mice

Gαi2^−/−^ deficient (Gαi2^−/−^) mice on a pure 129SvEv background were bred and maintained under specific pathogen-free conditions in microisolator cages with filtered air at the Department of Experimental Biomedicine, University of Gothenburg. Heterozygous Gαi2^+/−^ males were bred with heterozygous females, and the offspring were genotyped by polymerase chain reaction (PCR) analysis using genomic DNA from the tail. Homozygous Gαi2^−/−^ mice on this background develop a lethal colitis between the age of 5 and 8 weeks. In this study, mice were euthanized at 4–5 weeks (pre-colitic) or 5–9 weeks (colitic) and the absence/presence of colitis was confirmed post mortem. The experiments included both male and female mice as gender differences in clinical disease or immune parameters have not been found [7]. Both homozygous wild-type (WT) Gαi2^+/+^ and heterozygous (Gαi2^+/−^) mice were used as controls in the study (collectively referred to here as “WT mice”). RAG2^−/−^ mice on a 129SvEv background (a kind gift from F. Powrie, Univ. of Oxford) were bred homozygous, and were used as recipients in our cell transfer model.

### Media and antibodies

Fetal calf serum (FCS), 2-mercaptoethanol and Triton X-100 were purchased from Sigma-Aldrich (St. Louis, MO). Fixation/Permeabilization solution was purchased from eBioScience (San Diego, CA). Calcium and magnesium free HBSS (CMF-HBSS), HEPES, RPMI 1640 medium, GlutaMAX I and gentamicin were purchased from Invitrogen-GIBCO (Carlsbad, CA).

Antibodies used were: rat IgG2b anti-mouse CD16/CD32 (2.4G2), rat IgG2a anti-mouse CD4-Pacific Blue (PB) or -FITC (RM4-5), hamster IgG3 anti-mouse CD3-PE (145-2c11), CD62L (L-selectin/LECAM-1/Ly-22), CD44 (Pgp-1/H-CAM/Ly-24), rat IgG1 anti-mouse CD25-APC (PC61), rat IgG2a anti-mouse CD4 (L3T4), rat IgG2a κ isotype control, all BD Pharmingen (San Jose, CA); rat IgG2a isotype control (eBR2a), rat IgG2a anti-mouse Foxp3-PE or FITC (FJK-16S) (Flow Cytometry) or unconjugated (Immunohistochemistry), all eBioScience (San Diego, CA); goat anti-rat IgG Alexa Fluor 488, goat anti-rat IgG Alexa Fluor 568 amplification, all Molecular Probes-Invitrogen (Eugene, OR). Soluble α-CD3 mAb (10% supernatant from hybridoma 45-2C11) was produced in-house.

### Immunohistochemistry

Mouse colons were removed intact and rolled around a toothpick with proximal colon on the inside and distal colon on the outside of the roll, embedded in OCT compound (Tissue-Tek®, Miles Inc., Elkhart, IN), frozen in liquid nitrogen-cooled isopentane and stored at −80°C. Frozen sections, 6 µm (sectioned on a Leica CM3050s cryostat (Leica Microsystems, Wetzlar, Germany)), were fixed in 100% cold acetone for 10 min prior to H&E or fluorescence staining. For FoxP3/CD4 double labelling, sections were post fixed in 4% buffered formaldehyde for 5 min and permeabilized using 0.5% Triton X-100 for 5 min. After fixation/permeabilization, sections were incubated with rat IgG2a anti-mouse FoxP3, or with rat IgG2a isotype control, followed by detection using goat anti-rat IgG Alexa Fluor 568. The samples were then incubated with rat anti-mouse CD4, followed by goat anti-rat IgG Alexa Fluor 488. The slides were mounted in 4′, 6-diamidino-2-phenylindole (DAPI)-containing mounting medium (Molecular Probes-Invitrogen) and visualized with a Zeiss Axioskop 2 microscope (Carl Zeiss Imaging, Thronwood, NY) and analyzed by Axiovision 4,5 software (Carl Zeiss Imaging) or Leica DFC 480 (Leica Microsystems GmbH, Germany). Numbers of CD4^+^ cells and CD4^+^FoxP3^+^ cells were counted, using Image J (Image Processing and Analysis in Java, National Institutes of Health, U.S.A), at 3–5 sites of the mucosa in distal colons from four colitic Gαi2^−/−^ mice and 4 WT controls; and at least one colonic lymphoid patch (CLP) in each of four WT control mice.

### Preparation of cell suspensions

Single cell suspensions were prepared from the whole thymus, spleen or mesenteric lymph nodes (MLN) as previously described [1], [7]. Splenic erythrocytes were lysed with ammonium chloride and cells were then washed in phosphate-buffered saline (PBS) containing 1% heat inactivated fetal calf serum (FCS). For isolation of colonic lamina propria lymphocytes (LPL), dissected colons were washed free of fecal contents by flushing with saline. They were then opened longitudinally, washed extensively in Hank's Balanced Salt Solution without Calcium and Magnesium (CMF-HBSS) supplemented with 15 mM HEPES and incubated for 5×15 min at 37°C, in CMF-HBSS containing 5 mM EDTA to remove epithelial cells and intraepithelial lymphocytes. The remaining tissue was incubated for 15 min in RPMI 1640 containing 15 mM HEPES and 10% heat inactivated FCS, followed by three successive 60 min incubations in Liberase/blendzyme 3 (0.2 Wünsch units/ml, Roche Applied Science, Indianapolis, IN) in RPMI 1640 medium+GlutaMAX I containing 15 mM HEPES and 20% heat inactivated horse serum, yielding LPLs.

### Flow cytometry

Single cell suspensions of 1×10^6^ cells/30 µl were incubated on ice for 10 min with unconjugated anti-CD16/CD32 and then for 30 min with: anti-CD4-PB or -FITC; anti-CD3ε-PE; anti-CD25-APC; and anti-Foxp3-PE or -FITC.

For intracellular staining, cells were first permeabilized using Fixation/Permeabilization solution. Cells were then acquired on a BD LSRII flow cytometer (BD Biosciences, San Jose, CA), and the data analyzed using the Flow-Jo software (Tree Star Inc., San Carlos, CA).

### Cell fractionation

Splenic CD4^+^ cells were partially purified by negative selection on AutoMACS (Miltenyi Biotec, Bergisch Gladbach, Germany), following the manufacturer's instructions, using the mouse CD4^+^ T cell isolation kit (Miltenyi Biotec). Cells were then labeled with fluorochrome-conjugated CD4-PB, CD25-APC, CD3ε-PE in PBS supplemented with 2% FCS and 2 mM EDTA, washed and subsequently sorted into CD25^+^ and CD25^−^ subpopulations using a FACSAria® (BD Biosciences). Purity of the sorted cells (CD3^+^CD4^+^CD25^+^ or CD3^+^CD4^+^CD25^−^) ranged from 95% to 98%. The CD25^+^ and CD25^−^ populations contained 89.3±6.9% (n = 9) and 6.3±1.8% (n = 9) (Gαi2^−/−^ mice) or 91,0±6,0% (n = 11) and 4,3±1.4% (n = 6) (WT mice) FoxP3^+^ lymphocytes, respectively.

### Macroscopic scoring of colitis

Colonic pathology was macroscopically scored in a blinded fashion by two independent observers and graded for the following parameters: thickness (0–2), stiffness (0–3), edema (0–3) and visible ulcerations (0–1), with a total maximum score of 9 [22].

### Adoptive transfer

Freshly sorted splenic CD4^+^CD25^+^ (Treg) and CD4^+^CD25^−^ cells (Teff), either 4×10^5^ Teff only, or co-transferred with 1×10^5^ Treg, from colitic Gαi2^−/−^ and WT mice were adoptively transferred into 129SvEv RAG2^−/−^ mice by i.p. injection. RAG2^−/−^ mice on this background are resistant to colitis induction by WT effector T cells in the absence of *H hepaticus* infection [23].

### Analysis of cell division

Analysis of cell division by dye dilution was performed using a Vybrant CFDA SE cell tracer kit (CFSE) (Molecular Probes-Invitrogen). Effector T cells were washed twice with cold PBS, resuspended in 1 ml 5 µM CFSE solution/10^7^ cells, and incubated for 8 min at room temperature in the dark. Staining was quenched by adding an equal volume of FCS. Cells were then washed twice with complete culture medium and used in the suppression assay. The calculation of percent cell division of Teff within the Teff∶Treg co-cultures was performed using FlowJo software (Tree Star Inc., San Carlos, CA). The percentage of the CFSE-labelled Teff cells of the original sample which had divided are denoted as “Cell division %” in the graph. I.e., if all the Teff in the starting population have divided, the Cell division % = 100%.

### In vitro suppression assay

Sorted splenic CD4^+^CD25^−^ cells (Teff), 5×10^4^ cells per well, stained with CFSE, from colitic Gαi2^−/−^ or WT mice were incubated with CD4^+^CD25^+^ putative Treg from colitic Gαi2^−/−^ or WT mice at Teff∶Treg ratios of 2∶1, 4∶1, 8∶1 or Teff alone in 96-well round-bottom plates for 72 h at 37°C in 5% CO_2_ in the presence of soluble α-CD3 mAb in Basal Iscove complete medium containing 10% heat-inactivated fetal calf serum, 50 µM 2-mercaptoethanol, 1 mM L-glutamine, and 50 µg/ml gentamicin. Irradiated (25 Gy Cesium-137) T cell-depleted spleen cells from WT mice were used as accessory cells at 2×10^5^ cells per well.

### Cytokine measurements in supernatants

Cytokine concentrations in supernatants from the suppression assays were measured using a cytometric bead array (CBA) kit, mouse Th1/Th2/Th17 cytokine kit (BD Pharmingen); according to the manufacturer's instructions. Cytokine levels were quantitated using calibrated standards of each cytokine and analyzed by the CBA software.

### Statistical analysis

The Mann-Whitney non-parametric test for unpaired data, the Student t-test or one-way ANOVA (Tukey's Multiple Comparison Test) were used for comparison between groups. Differences were considered statistically significant at p<0.05.

## Results

### FoxP3 expression is restricted to CD4^+^ T cells

The localization of FoxP3 expression within T cell subpopulations in Gαi2^−/−^ and WT mice was analyzed, using mesenteric lymph node (MLN) cells. As illustrated in Table 1, FoxP3^+^ cells were restricted to the CD4^+^ T cell population in the MLN in both precolitic and colitic mice, with 80 to 90% residing in the CD4^+^CD25^+^ T cell population. The CD8^+^ T cell population had almost no expression of CD25 (<1% positive cells, data not shown) and Foxp3 expression was detected on less than 2% of CD8^+^ T cells [Table 1].

**Table 1:** Frequency of FoxP3^+^T cells in CD4^+^, CD4^+^CD25^+^and CD8^+^ subpopulations from mesenteric lymph nodes (MLN), as determined by flow cytometry.

			Mean % ± SD of FoxP3^+^	Range
CD4^+^	early colitic mice	n = 7	10.7±2.5	(7.34–13.3)
	control mice †	n = 7	9.0±1.0	(7.84–10.2)
	colitic mice	n = 7	11.4±1.6	(8.84–12.9)
	control mice ††	n = 7	9.1±1.4	(7.76–11.9)
CD4^+^CD25^+^	early colitic mice	n = 7	81.0±8.7	(71.1–94.6)
	control mice †	n = 7	82.0±11.9	(66.0–95.1)
	colitic mice	n = 7	79.2±5.7	(73.2–87.8)
	control mice ††	n = 7	91.2±2.2	(88.8–93.4)
CD8^+^	early colitic mice	n = 7	1.6±1.4	(0.29–3.73)
	control mice †	n = 7	1.2±1.0	(0.29–2.46)
	colitic mice	n = 7	0.6±0.3	(0.26–0.96)
	control mice ††	n = 7	0.4±0.2	(0.29–0.75)

† : 4–6 weeks old mice,

†† : 7–9 weeks old mice.

### Frequency of FoxP3^+^ lymphocytes in central, peripheral and gut-associated lymphoid tissues

**Figure 1:**
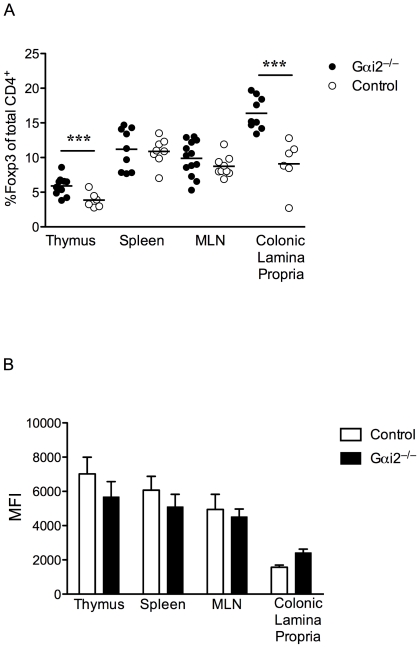
Frequency and intensity of expression of FoxP3 in CD4^+^ T cells in central, peripheral and mucosal lymphoid tissue. A. Frequencies of FoxP3^+^ cells in gated CD4^+^ T lymphocytes in colitic Gαi2^−/−^ mice were compared to age-matched control mice, (n≥6 in each group) as determined by FACS analysis. Each symbol represents one mouse and the mean of each group is indicated by a line. Results are shown from at least three independent experiments, where *** = p≤0.001. B. Intensity of FoxP3 expression, measured as median fluorescence intensity (MFI) of gated CD4^+^FoxP3^+^ lymphocytes. Results are shown as mean values ± SD of n = 4 to 6 mice per group, pooled from at least three independent experiments.

We and others have reported that precolitic Gαi2^−/−^ mice develop thymic atrophy, a significant decrease in the number of thymocytes, and a higher frequency of mature thymocytes, compared to WT mice [7], [24]. As FoxP3 expression is restricted to CD4^+^ mature single positive thymocytes, we examined the frequencies of CD4^+^FoxP3^+^ thymocytes in Gαi2^−/−^ compared to WT mice. Gαi2^−/−^ CD4^+^ mature thymocytes contained a significantly higher frequency of FoxP3^+^ cells compared to WT thymocytes (p<0.01) (Fig. 1A). In contrast, there were no significant differences in the frequency of splenic and MLN CD4^+^FoxP3^+^ T cells between colitic Gαi2^−/−^ and age-matched WT mice. The frequency of CD4^+^FoxP3^+^ T cells was, however, significantly higher (p<0.001) in the colonic lamina propria (LP) of colitic Gαi2^−/−^ mice, compared to the healthy colon of WT controls. In addition, we also analyzed the intensity of expression of FoxP3 within CD4^+^FoxP3^+^ cells in the thymus, spleen, MLN, and colonic lamina propria, measured as median fluorescence intensity (MFI). No statistically significant differences were found in these four lymphoid tissues between colitic Gαi2^−/−^ and age-matched WT mice, (Fig. 1B). These data indicate that the spontaneous chronic inflammation in Gαi2^−/−^ mice cannot be explained by a simple quantitative reduction of Treg within total colonic tissues.

**Figure 2:**
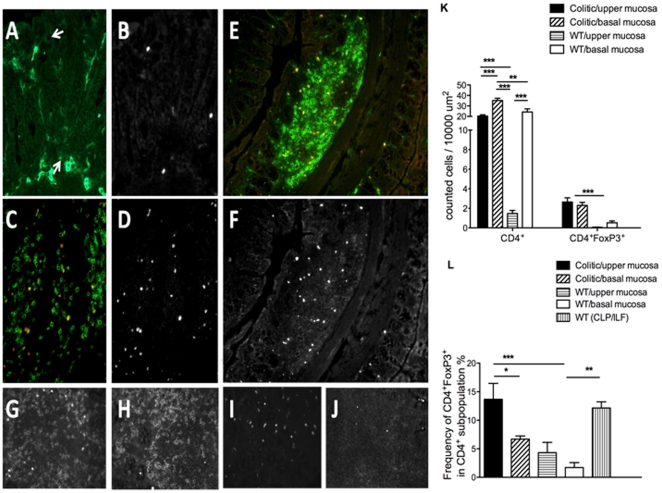
Immunohistochemical analysis of distribution of CD4^+^FoxP3^+^ cells in colonic tissues. A, B, E, F = colon from WT mice; C, D = colon from Gαi2^−/−^ mice with colitis. Tissues were double stained for CD4 and FoxP3. Images shown are double positive (DP) CD4^+^ (green) FoxP3^+^ (red) (A, C, E) (where DP cells appear as yellow), or single positive (SP) FoxP3 (grey) (B, D, F); In WT colonic mucosa (A, B), CD4^+^ T cells were few in number and were mostly confined to the basal lamina propria. In colitis (C, D), CD4^+^ T cells infiltrated the entire lamina propria and included many DP cells. Within colonic lymphoid patches (CLP) of WT mice (E, F), interfollicular T cell areas contained many DP CD4^+^FoxP3^+^ cells (compare to paucity of DP cells in normal mucosa to left and right). G–J, controls: G, CD4/FoxP3; H, CD4/FoxP3 isotype control; I, CD4 isotype control/FoxP3; J, CD4 isotype control/FoxP3 isotype control. Original magnifications: ×20. K. Absolute numbers of CD4^+^ cells and CD4^+^FoxP3^+^ cells were counted at 3–5 sites of mucosa in distal colons from 4 colitic Gαi2^−/−^ mice and 4 WT control mice; at least one Colonic Lymphoid Patches (CLP) was counted in 4 WT control mice. “Upper” and “lower” lamina propria were defined as above crypt bases and between crypt base and muscularis mucosa, respectively. L. Frequency of FoxP3^+^ cells within the CD4^+^ population, as calculated from the results in K. Results are shown as mean values ± SEM of n = 4 mice per group, where * = p≤0.05, ** = p≤0.01 and *** = p≤0.001.

However, as it is likely that the function of Treg is related to their distribution within tissues in relation to effector T cells, we further examined their tissue distribution in colonic tissues of WT and colitic Gαi2^−/−^ mice, by immunohistochemistry. In the lamina propria of WT mice, CD4^+^ T cells were sparse, mostly restricted in the tissue to the level of the crypt bases, and contained few FoxP3^+^ cells (1–4%, Fig. 2A–B, K–L). In colonic lymphoid patches (CLP) of WT mice, large numbers of CD4^+^FoxP3^+^ Treg were observed, restricted to the interfollicular T cell areas (Fig. 2E–F), comprising 10–15% of the total CD4^+^ T cells in this tissue (Fig. 2L). In colitic Gαi2^−/−^ mice, the distribution was quite different. In the inflamed colon, no discrete CLP with associated clustered CD4^+^FoxP3^+^ Treg were observed. Instead, the colonic lamina propria, particularly above the level of crypt bases, was heavily infiltrated by CD4^+^ cells – a 10× increase over equivalent WT tissues (Fig. 2A–J, K). A significant number of these CD4^+^ infiltrating cells expressed FoxP3 (Fig. 2K), with a 10–20× increase in the number of CD4^+^FoxP3^+^ cells in the basal and upper mucosa, respectively. Similar to the flow cytometric analysis, their frequency in the upper lamina propria were increased 2–3× compared to CD4^+^ cells in WT colon (Fig. 2L). Thus, the hyperplastic mucosa in established colitis was heavily infiltrated by a ten-fold increase in CD4^+^ T cells, and the proportion of FoxP3^+^ cells rose by two- to three-fold. However, this was preceded by a loss of CLP, which, in the WT colon, contain the major proportion of Treg.

### High frequencies of CD103-expressing Treg in colitic Gαi2^−/−^ mice

**Figure 3:**
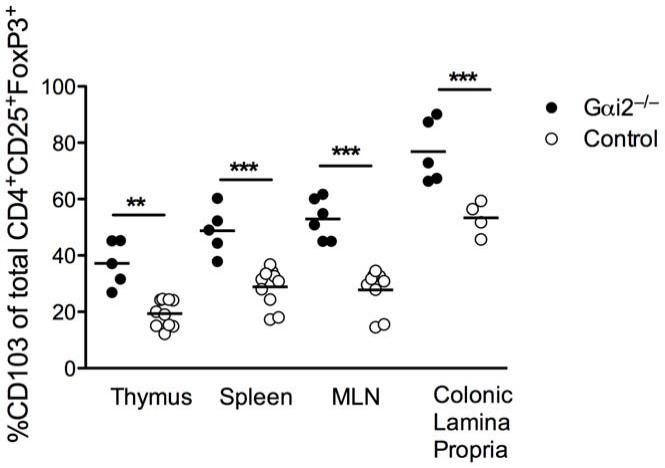
Expression of integrin αE (CD103) on CD4^+^CD25^+^FoxP3^+^ Treg cells from central, peripheral and mucosal lymphoid tissue. Frequencies of CD4^+^CD25^+^FoxP3^+^ Treg cells in thymus, spleen, mesenteric lymph nodes (MLN) and colon from 5 to 8 weeks old colitic Gαi2^−/−^ mice and age-matched WT littermates. (n≥6 in each group), expressing CD103 were determined by FACS analysis. Gates were set on the CD4^+^CD25^+^FoxP3^+^ subpopulation of lymphocytes. Each symbol represents one mouse and the mean is indicated by a line. Results pooled from at least three independent experiments, where ** = p≤0.01 and *** = p≤0.001.

Integrin αE(CD103)ß7 contributes to mucosa specific retention of T cells and other cells within epithelia by binding to E-cadherin [25], [26]. In addition, CD103 has a possible accessory function for activation of intraepithelial lymphocytes. It has been reported that CD103 is a marker for activated Treg [27] We therefore investigated the frequency of Treg with CD103 expression in primary, secondary and gut-associated lymphoid tissues in Gαi2^−/−^ mice. As shown in Fig. 3, the frequency of CD4^+^FoxP3^+^ T cells expressing CD103 was significantly increased in Gαi2^−/−^ compared to WT mice in all four tissues studied: thymus, spleen, MLN, and colonic lamina propria.

### Treg from colitic Gαi2^−/−^ mice fail to protect against colitis in a transfer model

**Figure 4:**
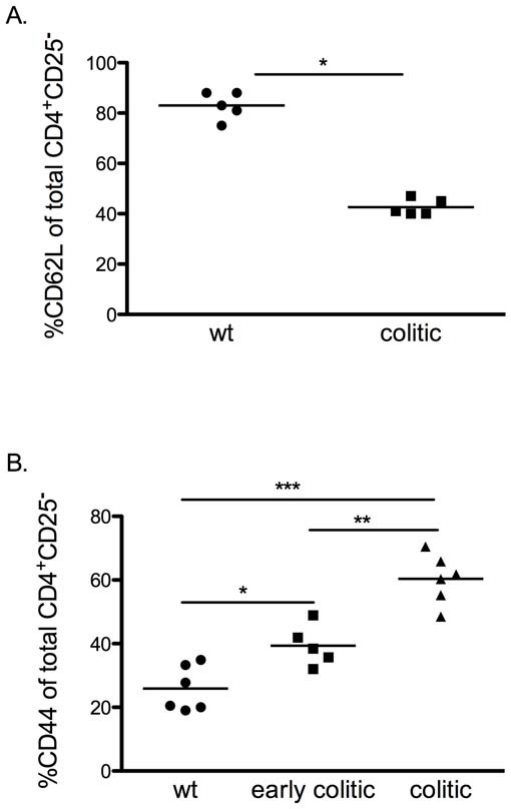
Increased frequencies of CD62L^+^ and CD44^+^ effector T cells in spleens of Gαi2^−/−^ mice. A. Frequencies of CD62L^+^ cells in gated CD4^+^CD25^−^ splenocytes in colitic Gαi2^−/−^ mice were determined by FACS analysis and compared to age-matched control mice, (n = 5 in each group) B. Expression of CD44^+^ cells in gated CD4^+^CD25^−^ splenocytes in early colitic, colitic Gαi2^−/−^ mice and age-matched control mice, (n = 5–6 in each group). Each symbol represents one mouse and the mean of each group is indicated by a line. Results are shown from at least three independent experiments, where * = p≤0.05, ** = p≤0.01 and *** = p≤0.001.

**Figure 5:**
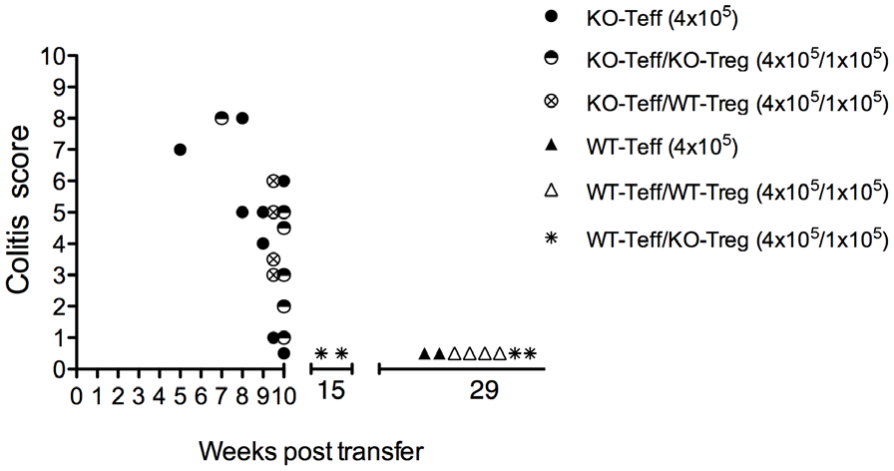
Treg from colitic Gαi2^−/−^ mice fail to protect against colitis in a transfer model. Freshly isolated and sorted donor splenic CD4^+^CD25^−^ Teff cells from colitic Gαi2^−/−^ mice (KO-Teff) or CD4^+^CD25^−^ effector T cells from WT mice (WT-Teff) were transferred with or without splenic CD4^+^CD25^+^ Treg from Gαi2^−/−^ mice with colitis (KO-Treg) or WT CD4^+^CD25^+^ Treg (WT-Treg) at a 4∶1 (Teff∶Treg) ratio into RAG2^−/−^ recipients by i.p. injection. The results are based on three separate experiments.

Our group and others have reported that CD4^+^ T cells from Gαi2^−/−^ mice undergo altered development and amplified responses to TCR ligation [7], [8]. The increase in frequency of Treg among total CD4^+^ T cells in the thymus and in the inflamed colon of Gαi2^−/−^ mice, compared to WT mice prompted us to investigate the functional activity of the Treg in vivo and in vitro. CD4^+^CD25^−^ effector T cells from colitic Gαi2^−/−^ mice (KO-Teff), or CD4^+^CD25^−^ effector T cells from WT mice (WT-Teff) were transferred with or without CD4^+^CD25^+^ Treg from Gαi2^−/−^ mice with colitis (KO-Treg) or WT mice (WT-Treg) at a 4∶1 Teff∶Treg ratio into RAG2^−/−^ recipients. Analysis of the activation phenotype of these transferred CD4^+^CD25^−^ cells showed that those derived from colitic Gαi2^−/−^ donor spleens contained a significantly higher proportion of cells with a CD4^+^CD44^+^CD62L^−^ effector memory phenotype than those derived from WT spleens. Interestingly, the data also show that even in mice of three weeks of age, before the development of colitic symptoms, splenic CD4^+^ T cells contain significantly higher proportions of effector memory cells (Fig. 4). Five to seven weeks after T cell transfer, mice receiving WT-Teff cells did not develop disease, irrespective of the presence or absence of Treg from either donor. These mice were observed up to 15 or 29 weeks after transfer and did not develop colitis within this time frame (Fig. 5). This is consistent with previous studies demonstrating that, in contrast to the SCID or RAG transfer models on backgrounds other than 129SvEv, in which naïve wild type CD45RB^hi^ T cells induce colitis upon transfer [21], RAG^−/−^ mice on a 129SvEv background, are resistant to colitis induction by wild type cells in the absence of *H. hepaticus* infection [23].

However, the majority of mice (6/8) receiving KO-Teff cells alone in two separate experiments developed clinical signs of disease (Fig. 5). Co-transfer of KO-Treg or WT-Treg at a 4∶1 Teff∶Treg ratio at 4×10^5^ had little effect on induction of pathology (5/6 and 4/4, respectively, developing colitis). Thus, the KO-Teff cells were highly pathogenic and the pathology induced was unaffected by co-transfer of Treg from either WT or Gαi2^−/−^ mice.

### Treg from Gαi2^−/−^ mice are fully functional *in vitro*

**Figure 6:**
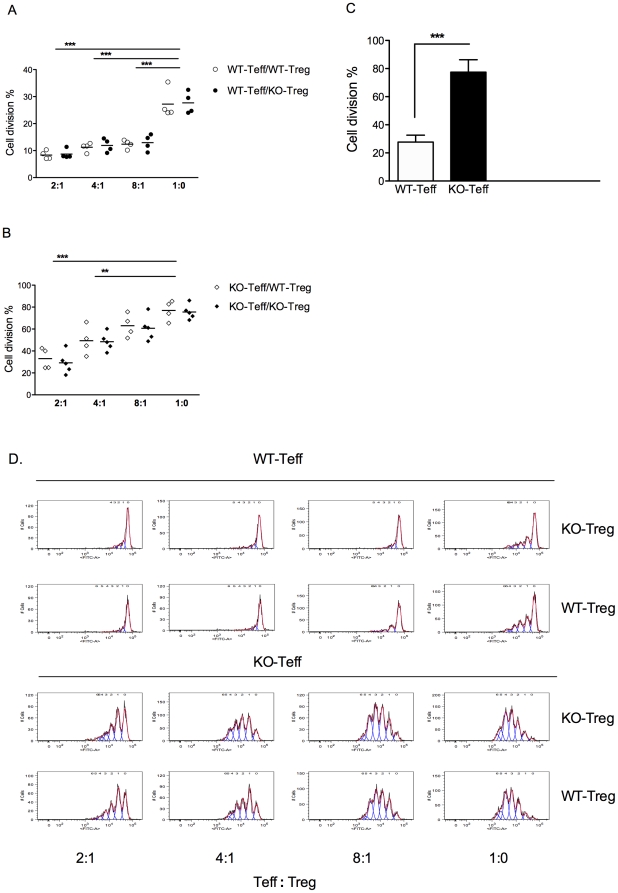
Treg from Gαi2^−/−^ mice are fully functional *in vitro*, but KO-Teff are less readily suppressed than WT-Teff. CD4^+^CD25^−^ effector T cells (Teff) and CD4^+^CD25^+^ regulatory T cells (Treg) were sorted from the spleen of 5 to 8 weeks old colitic Gαi2^−/−^ mice and from age-matched WT littermates, respectively. These effector and regulator cells were mixed at various ratios and in different combinations. CD4^+^CD25^−^ Teff cells were pre-stained with CFSE before being added to co-cultures. A–B. In the suppression assay, Gαi2^−/−^ Treg (KO-Treg) or WT Treg (WT-Treg) were added at the indicated ratios with either Gαi2^−/−^ Teff (KO-Teff) or WT Teff (WT-Teff) (5×10^4^ cells per well). The mixtures of cell populations were co-cultured with irradiated T cell depleted splenocytes from WT mice as accessory cells (2×10^5^ cells) and soluble α-CD3 mAb for 72 hours. Means ± SEM of cell divisions in the suppression assay from FACS analysis is shown. The results presented are from 4 independent experiments, where ** = p≤0.01 and *** = p≤0.001. C. Means ± SEM of cell divisions of CFSE WT-Teff and KO-Teff from the wells with Teff only, was analyzed by FACS analysis. The results are based on 4 independent experiments, where *** = p≤0.001. D. Representative histograms showing the division of CFSE labeled cells in the absence or presence of KO-Treg or WT-Treg.

The apparent insufficient regulatory function demonstrated in the in vivo cell transfer model of colitis raises questions regarding the inherent function of the Treg population in the Gαi2-deficient immune system. It has been shown that co-culture of CD4^+^CD25^+^ Treg with CD4^+^CD25^−^ effector T cells (Teff) from wild-type mice results in inhibition of the proliferative response of CD4^+^CD25^−^ effectors [28]. We therefore established an in vitro suppression assay with mixed combinations of Treg and Teff from Gαi2^−/−^ and WT mice at different ratios. Regulation of these Teff responses were analyzed in Teff∶Treg co-cultures by cell cycle analysis, using CFSE labeling (Fig. 6). It is apparent from the cell division data (Fig. 6A, B, D) that Treg from both WT (WT-Treg) and Gαi2^−/−^ mice (KO-Treg) were able to suppress proliferation of both WT-Teff and KO-Teff, with the former being more readily suppressed (even at 8∶1) than KO-Teff which were suppressed less readily; (at a 4∶1 ratio) (Fig. 6A–B). Thus, the in vitro suppressive functions of the Treg populations from the two strains of mice were equivalent. However, this again highlights that KO-Teff were more responsive to signaling through CD3, probably reflecting their pathogenic potential. This was confirmed by comparison of proliferation of the Teff populations alone, measured by CFSE cell cycle analysis (Fig. 6C), demonstrating significantly higher percentages of WT Teff undergoing cell division compared to KO Teff.

**Figure 7:**
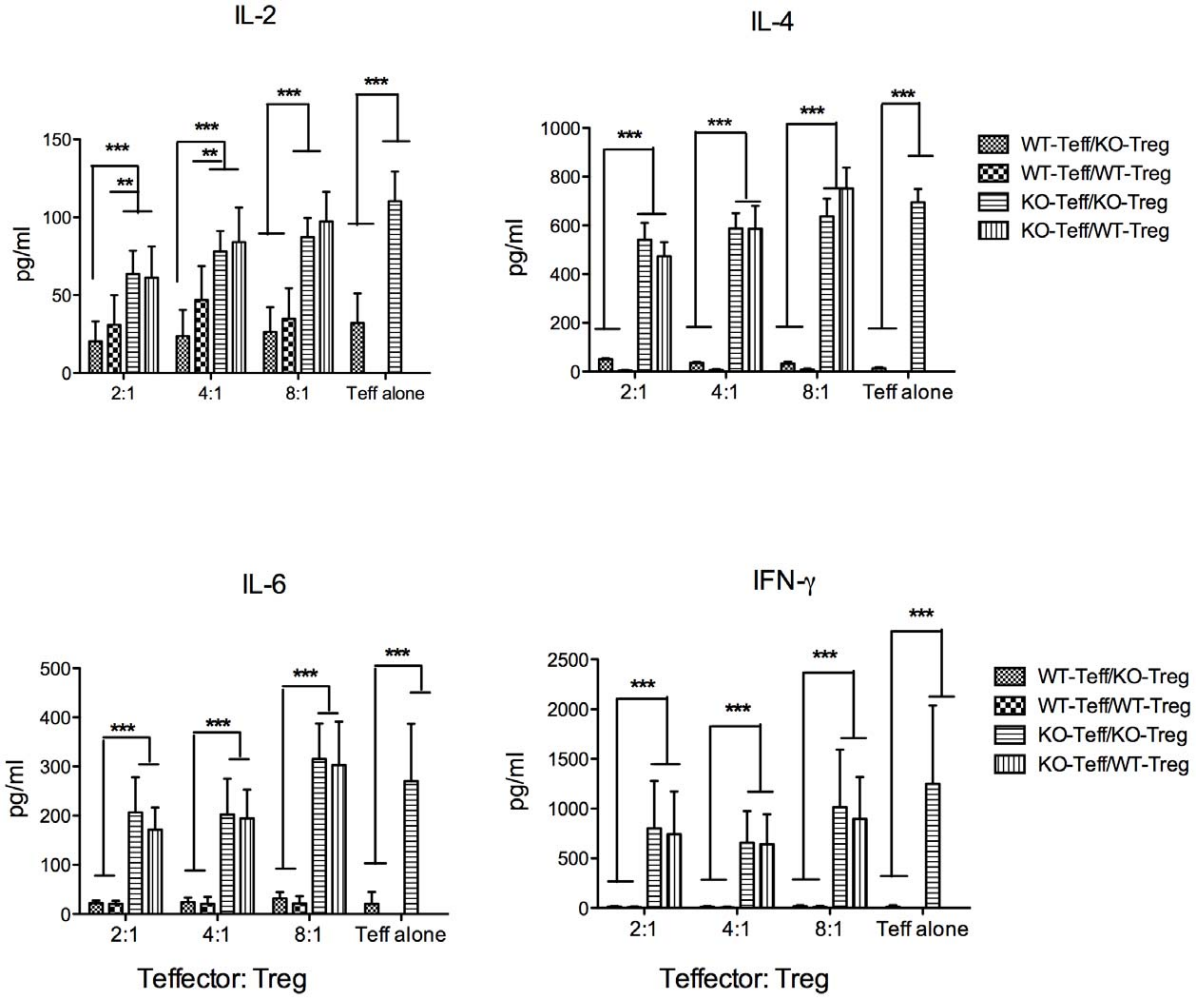
Cytokine production in the suppression assay co-cultures. Supernatants were harvested after 48 hours and assayed for IL-2, IL-4, IL-6 and IFN-γ by CBA analysis. Means ± SEM of cytokine levels from the collected supernatants in two of the triplicate wells are shown. Results presented are from 3 independent experiments, where ** = p≤0.01 and *** = p≤0.001.

Finally, an assessment of Teff cytokines produced in these cultures was made. Supernatants of anti-CD3-activated WT Teff contained relatively low levels of IL-2, IL-4, IL-6, or IFN-γ and it was not possible to define any significant effects on cytokine levels mediated by either WT-Treg or KO-Treg (Fig. 7). However, significantly higher levels of cytokines were measured in supernatants of cultures containing activated splenic KO-Teff compared to WT-Teff: 2–3-fold higher for IL-2; 20–60-fold higher for IL-4; 10–20-fold higher for IL-6; and up to 130-fold higher for IFN-γ (Fig. 7). It was not possible to define any significant reductions in effector cytokines attributable to addition of Treg to the co-cultures, although a trend towards reduced cytokine production with increasing amounts of Treg in the cultures was noticed. The cytokines IL-10, IL-17 and TNF-α were below the detection limit of our assay.

## Discussion

Both nTreg and iTreg play a critical role in maintenance of peripheral tolerance by suppressing potential autoimmune responses, while simultaneously regulating inflammatory responses to microbes. Our experiments show that FoxP3-expressing Treg in Gαi2^−/−^ mice are restricted to the CD4^+^ subpopulation and that these CD4^+^FoxP3^+^ T cells are found in higher frequencies in the thymus and colon of colitic Gαi2^−/−^ mice.

The high frequency of CD4^+^FoxP3^+^ thymocytes is consistent with the Gαi2 deficient mice having abnormal thymocyte development resulting in an increase in positive selection and aberrant development of CD4^+^CD25^+^ T regulatory cells [24]. No differences in intensity of FoxP3 expression by CD4^+^ T cells were found in the thymus, spleen, MLN and colonic lamina propria of colitic Gαi2^−/−^ mice and age-matched controls, indicating that the phenotype is consistent in both groups.

In the colon, the proportion of isolated CD4^+^ cells which expressed FoxP3 increased 2–3-fold, compared to WT mice. This might be expected if these putative Treg are migrating into an established site of inflammation in an attempt to restore homeostasis, a phenomenon that has also been observed in rheumatoid arthritis patients [29]. However, it has been suggested that Treg are generated within special DC-containing structures within the colon in response to infiltration by pathogenic CD4^+^ T cells [30], implying either a structural reservoir of Treg precursors within the colon, or a colonic organized lymphoid structure in which Treg may be generated via DC interactions. To examine structural implications, we derived distribution data using immunohistochemistry. These data show that in the WT mice, the overwhelming majority of colonic FoxP3^+^ Treg, are indeed associated with organized CLP (and isolated lymphoid follicles (ILF)) structures. In accordance with our previous demonstration of regression of Peyer's patches in Gαi2^−/−^ mice prior to colitis [2], CLP and ILF were not observed in inflamed Gαi2^−/−^ colons but the numbers of lamina propria CD4^+^FoxP3^+^ cells increased in inflammation, with their frequency within the total CD4^+^ population increasing, based on both flow cytometry and immunohistochemistry data. We speculate that CLP represent the WT equivalent of the minimal DC structures seen in the RAG2^−/−^ transfer model [30] and that they may constitute reservoirs in which Treg either accumulate and/or are generated, although this requires further investigation. Regardless of this interpretation, CD4^+^ cells with a Treg phenotype clearly accumulate in significantly increased frequency within the inflamed colonic mucosa.

It is possible that the higher frequency of FoxP3^+^ cells in the colonic lamina propria of colitic Gαi2^−/−^ mice may be a consequence of ongoing inflammation, as there was no significant difference in the frequency of CD4^+^FoxP3^+^ T cells at other peripheral sites, such as spleen and MLN, between Gαi2^−/−^ and WT mice. These findings concur with reports from human disease - in patients with active IBD, the number of FoxP3^+^ cells in the affected intestinal mucosa correlates with disease activity [31], [32].

Our data show that CD4^+^FoxP3^+^ Treg, in both central and peripheral lymphoid tissue in colitic Gái2^−/−^ mice have a substantially higher frequency of cells expressing CD103, compared to controls. CD103 have been reported to be an activation marker for Treg [33]. The CD4^+^FoxP3^+^CD103^+^ subset expresses CTLA-4; suppresses T cell proliferation *in vitro*; and protects mice from colitis in the severe combined immunodeficient (SCID) transfer model *in vivo* [33]. Integrin αE(CD103)β7 can therefore be regarded as a marker for activated regulatory T cells, including those operating at the mucosal barrier. It is possible that in the steady state, Treg efficiently home to lymph nodes, expand and accumulate where cognate antigens are present. This basal level of activity prevents priming of autoreactive T cells and maintains immune homeostasis. However, when this first line of protection fails and barrier breakdown, tissue destruction and inflammation occur, changes in the local microenvironment enable further activation of T cells into Treg with high expression of CD103, allowing their traffic to affected tissues [34].

We have shown that Treg from colitic Gαi2^−/−^ mice are as potent as WT Treg in inhibiting the proliferative response of both WT-Teff and KO-Teff following polyclonal TCR-mediated stimulation *in vitro*, although KO-Teff were less inhibited by either Treg population compared to WT-Teff. The crucial finding of our experiments is that Gαi2^−/−^ Treg are fully functional and there is no endogenous Gαi2-related defect. Nonetheless, these apparently potent Gαi2^−/−^ Treg, paradoxically, were only partially protective against colitis in the RAG2^−/−^ transfer model. However, the protective effect of Gαi2^−/−^ Treg *in vivo* was comparable to WT Treg. On the basis of the *in vitro* and *in vivo* data, we argue that the dysregulated immune activity in this model is a product of heightened effector cell activity, and not of reduced Treg function, within the colitic lesion.

Our present and previous data by others clearly demonstrate that T effectors from Gαi2^−/−^ mice are more pro-inflammatory [8]. This is presumably a reflection of the phenotype of the cells we have used as effectors. The Gái2^−/−^ Teff population comprised many more cells with a CD4^+^CD62L^−^ CD44^+^ effector memory phenotype than WT Teff, which are more easily activated to proliferate and less susceptible to regulation by Treg. In addition, IL-6, one of the proinflammatory cytokines secreted at high levels by KO-Teff in this study, has been shown to enhance the resistance of T effector cells to the suppressive effects of Tregs [35], [36]. With regard to this possibility, administration of IL-6R mAb to SCID mice, after transfer of CD45RB^high^ T cells, confers protection from colitis [37], and IL-6–deficient mice have been shown to be less susceptible to colitis [38]. Finally, cells with this phenotype can actively suppress the induction of iTreg by cell contact and secreted factor-mediated mechanisms [39]. Even in young mice, before the age of onset of frank colitis, Gái2^−/−^ CD4^+^ T cells are pushed towards this phenotype. It seems likely, then, that the Gái2^−/−^ deletion drives the differentiation of CD4^+^ T cells towards this easily activated, not readily regulated, effector memory phenotype, which proliferate to bacterial antigens in the Gái2^−/−^ colon with a potentially defective epithelial barrier [5], and actively suppress the induction of appropriate numbers of inducible regulatory T cells [39]. In addition, defective chemokine receptor signaling within the Gαi2^−/−^ environment may reduce the infiltration of effective numbers of Treg.

In conclusion, Gαi2^−/−^ Treg that are functionally active *in vitro* are enriched in the inflamed colon in Gαi2^−/−^ mice, but are unable to regulate the highly potent Gαi2^−/−^ effector T cells, which we demonstrated to be significantly more resistant to suppression by either WT or Gαi2^−/−^ Treg, compared to WT effector T cells. Further studies are in progress to investigate the relative kinetics of mucosal Teff and Treg infiltration during pathogenesis.
